# Price, Income, and Affordability as the Determinants of Tobacco Consumption: A Practitioner’s Guide to Tobacco Taxation

**DOI:** 10.1093/ntr/ntaa134

**Published:** 2020-07-22

**Authors:** Nigar Nargis, Michal Stoklosa, Ce Shang, Jeffrey Drope

**Affiliations:** 1 Economic and Health Policy Research, American Cancer Society, Inc., Atlanta, GA; 2 Department of Internal Medicine, The Ohio State University Wexner Medical Center, Columbus, OH

## Abstract

**Introduction:**

Tobacco product prices and consumers’ income are the two major economic determinants of tobacco demand. The affordability of tobacco products is dependent on the price of tobacco products relative to consumer income. Increase in tobacco tax is expected to lead to higher price, lower affordability, and reduced consumption. Price elasticity and affordability elasticity are used in analyzing the effect of tobacco tax increases on tobacco consumption and public health. The availability of both parameters raises the question of which one to apply in policy discussions.

**Aims and Methods:**

Using global data on cigarette consumption, price, income, and tobacco control measures for 169 countries over 2007–2016, this study estimated the price elasticity and affordability elasticity of cigarette consumption by country income classification using country-specific fixed effects model for panel data.

**Results:**

The estimates show that the restriction of equal strength of the effects of price and income changes on tobacco consumption maintained in affordability elasticity estimation is valid for low- and middle-income countries, while it is rejected for high-income countries.

**Conclusions:**

Affordability elasticity may prove to be a useful parameter to explain and predict the sensitivity of consumers to tobacco tax and price policy changes under conditions of robust economic growth, which are more likely to be observed in countries with initial low- or middle-income setting. It can provide a reasonable benchmark for tobacco tax and price increase necessary to effectively reduce affordability and consumption of tobacco, which can form a basis for building systematic tax and price increases into the tobacco tax policy mechanism.

**Implications:**

Price elasticity measures the sensitivity of consumers to changes in real prices, holding real income constant. Affordability elasticity measures the sensitivity of consumers to price changes adjusted for inflation and income changes. Existing scientific literature on tobacco demand abounds in both price and affordability elasticity estimates, without providing a clear explanation of the theoretical and policy implications of using one parameter over the other. By estimating and comparing price and affordability elasticities for high-income and low-and-middle-income countries separately, this article offers a guide to the practitioners in tobacco taxation for evaluating the effectiveness of tax-induced price increases on tobacco consumption.

## Introduction

Tobacco product prices and consumers’ income are the two major economic determinants of tobacco demand. Consumers’ purchasing power or affordability of tobacco products is dependent on the price of tobacco products relative to consumer income. Tobacco taxation, through its effect on tobacco product prices, influences the affordability of tobacco products and in turn consumers’ demand for tobacco products. Increase in tobacco tax is expected to lead to higher tobacco product price, make tobacco products less affordable, and reduce tobacco consumption. However, income growth can offset the effect of tax and price increases in reducing consumption by making tobacco products more affordable. Thus, for tobacco taxation to be an effective tobacco control measure, it is necessary that the effect of a tax-induced price increase in reducing tobacco consumption more than offsets the effect of income growth that can induce increases in tobacco consumption.

Classic economic modeling of tobacco demand estimates price and income elasticity separately to measure the effects of price and income changes on tobacco demand. Price elasticity measures the sensitivity of tobacco demand to changes in tobacco product prices after adjustment for inflation (real prices), holding real income and other factors constant. Income elasticity similarly measures the sensitivity of tobacco product demand to income changes, holding tobacco product real prices and other factors constant. A negative price elasticity of demand for tobacco products indicates that a price increase causes a reduction in tobacco consumption holding all else constant, including income. If income increases the demand for tobacco products, it is not guaranteed that tobacco consumption will decrease following a price increase because the net effect of simultaneous changes in price and income on tobacco product demand will depend on the relative strength of these two effects.

Affordability elasticity, on the other hand, measures the sensitivity of tobacco product demand to changes in the tobacco product price and income growth. A negative affordability elasticity would, therefore, imply that a price increase that outweighs the effect of income growth will lead to a reduction in tobacco consumption.

Existing literature on the economics of tobacco demand abounds in the estimates of price and income elasticities of demand, largely for cigarettes and to a limited extent for non-cigarette tobacco products (eg, smokeless tobacco) and electronic nicotine delivery systems (eg, e-cigarettes). Systematic reviews of these studies estimating price and income elasticities of tobacco product demand are available elsewhere.^1,2^

The estimates of affordability elasticity of tobacco products are becoming more prominent. A seminal study on men in Britain for the 25-year period 1946–1971 estimated the elasticity of cigarette demand with respect to price as a percentage of annual per capita personal disposable income, referred to as the “price–income ratio,” ranging from −0.44 to −0.58.^3^ This study also estimated price elasticity of cigarette ranging from −0.50 to −0.66.

The “price–income ratio” came to be formally known as the Relative Income Price (RIP) as a measure of affordability. The higher the value of RIP, the lower the affordability, and vice versa. Blecher and Van Walbeek^4^ used RIP given by the percentage of per capita gross domestic product (GDP) required to purchase 100 packs of cigarettes as a measure of the affordability of cigarettes. They investigated the relation between cigarette affordability and consumption by estimating the affordability elasticity of demand. Using data on 70 countries, they estimated that a 1% increase in the RIP was expected to decrease cigarette consumption by between 0.49% and 0.57%.

The estimation of affordability elasticity has become more salient for evaluating the effectiveness of tobacco tax increases as a tobacco control measure in line with the guidelines for implementation of Article 6 on price and tax measures to reduce the demand for tobacco under the World Health Organization Framework Convention on Tobacco Control (WHO FCTC).^5^ Zheng et al.^6^ obtained a national-level estimate of the affordability elasticity of cigarette consumption in China at −0.60 using data for the period 2001–2016. A more recent study estimated a conditional cigarette affordability elasticity of demand at −0.165 among Chinese adult smokers aged 45 and older.^7^ A global study by He et al.^8^ estimated affordability elasticity of cigarette consumption for 78 countries at −0.20 using data for the period 2001–2014.

Price elasticity and affordability elasticity are conceptually quite different. The availability of both price elasticity (in conjunction with income elasticity) and affordability elasticity leads one to ask which parameter is more appropriate in assessing the effectiveness of tobacco tax and price increases in reducing tobacco consumption. In this article, we explored the empirical and policy implications of using one elasticity over the other with the aim of providing a guide to the practitioners in tobacco taxation including researchers, tobacco control advocates, and policy makers.

## Methods

### Data and Measures

The primary outcome variable is annual cigarette consumption per adult (in number of cigarette sticks) calculated by dividing the total retail volume of cigarettes (available from the Euromonitor International Database) by the size of the adult population aged 15 and older (available from the US Census Bureau International Database) in each country and year from 2007 through 2016.^9,10^ The average price per pack of 20 cigarettes was calculated by dividing the total retail value by the annual retail volume. The nominal price variable for each country over time was adjusted for inflation and converted into 2016 constant prices (real prices) using the consumer price indices (CPIs) for respective countries. For cross-country comparability of prices, the real prices were converted into international dollars using 2016 purchasing power parity (PPP) conversion factors for respective countries. The CPI and the PPP conversion factors were obtained from the World Economic Outlook database of the International Monetary Fund.^11^ We used per capita gross domestic product (PGDP) as the income variable adjusted for inflation and PPP using annual GDP deflators and 2016 PPP conversion factors for respective countries and expressed in 2016 constant prices like the price variable. The PGDP data in current local currency units were drawn from the World Bank World Development Indicators (WDI) database.^12^

Following Blecher and Van Walbeek,^4^ the affordability of cigarettes was measured as the percentage of income required to purchase 100 packs of 20 cigarettes. The price and income variables as described above were used to construct the RIP variable. We controlled for aggregated country-level demographic and macroeconomic characteristics in the analysis. These variables include the percentage of working-age persons aged 15–64 (available from the US Census Bureau International Database), the percentage of females in the total population, and the percentage of the unemployed in the total labor force (available from the WDI database).^10,12^ In addition, we used the composite MPOWER scores to control for the tobacco control policy environment reflected in the level of implementation of the WHO FCTC. The WHO FCTC is the first international public health treaty under the auspices of the World Health Organization, which was adopted in 2003 and came into force in 2005 to protect nations from the devastating health, economic, social, and environmental consequences of tobacco use and exposure to tobacco smoke.^13,14^ In 2008, WHO introduced the package of six best-practice and cost-effective demand-reduction tobacco control policy measures contained in the WHO FCTC—monitor tobacco use (M); protect people from tobacco smoke (P); offer help to quit tobacco use (O); warn about the dangers of tobacco (W) with two subcomponents, health warnings (W1) and mass media (W2); enforce bans on tobacco advertising, promotion and sponsorship (E); and raise taxes on tobacco products (R)—collectively known as MPOWER.^15^ The country-specific MPOWER scores were systematically measured and reported in the biannual WHO Report on the Global Tobacco Epidemic since 2007.^15–20^ Countries were categorized into low- and middle-income countries (LMICs) and high-income countries (HICs) based on World Bank economic classification in 2016 available in the WDI database.^12^ More detailed information on data sources, construction of measures, and summary statistics is provided in Supplementary Appendix A.

### Analytical Framework

We used the following two model specifications to examine the relationship of cigarette consumption per adult (*C*) to price (*P*) and income (*Y*) controlling for observable country-specific characteristics (*X*), country-specific trends (*t*_*i*_), and unobserved country-specific heterogeneity (*α*_*i*_) that can explain variability in cigarette consumption.

$$\begin{array}{l} Model\;1:\ln C_{it}=\alpha_{0}+\alpha_{1}lnP_{it}+\alpha_{2}lnY_{it}+\alpha_{3}X_{it}+\alpha_{4}t_{i}+\alpha_{i}+u_{it} \end{array}$$ (1)

where $\alpha_{1}$ and $\alpha_{2}$ represent price and income elasticity of cigarette consumption. $\alpha_{1}$ is expected to be negative implying negative relationship between price and consumption by the law of demand. $\alpha_{2}$ is expected to be positive implying that tobacco product is a normal good and the demand for tobacco products increases with income growth.

$$\begin{array}{l} Model\;2\;:\;\;lnC_{it}=\;\;\;\beta_{0}+\beta_{1}ln\;\;\;RIP_{it}+\beta_{3}X_{it}+\;\;\;\beta_{4}t_{i}+\;\;\;\beta_{i}+e_{it} \end{array}$$

where $\;RIP_{it}=\;\frac{100\;\times \;P_{it}}{Y_{it}}$ and $\beta_{1}$ represents the affordability elasticity of cigarette consumption and the coefficients of the other control variables have the same interpretation as in equation (1). $\beta_{1}$ is expected to be negative implying that the higher the proportion of income required to purchase 100 packs of cigarettes, the lower the affordability and the lower the level of cigarette consumption.

Model 2 can be rewritten as:

$$\begin{array}{l} \begin{array}{ll} & InC_{it}=\;\;\;\beta_{0}+\beta_{1}ln\left(\frac{100\;\;\;\times \;\;\;P_{it}}{Y_{it}}\right)+\beta_{3}X_{it}+\;\;\;\beta_{4}t_{i}+\;\;\;\beta_{i}+e_{it}\\ & =\;\;\;\beta_{0}+\beta_{1}ln100+\beta_{1}lnP_{it}-\beta_{1}lnY_{it}+\beta_{3}X_{it}+\beta_{4}t_{i}+\;\;\;\beta_{i}+e_{it}\\ & =\;\;\;\left(\beta_{0}+4.61\beta_{1}\right)+\beta_{1}lnP_{it}+\beta_{2}lnY_{it}+\beta_{3}X_{it}+\;\;\;\beta_{4}t_{i}+\;\;\;\beta_{i}+e_{it}\\ & =\;\;\;\beta_{0}^{*}+\beta_{1}lnP_{it}+\beta_{2}lnY_{it}+\beta_{3}X_{it}+\;\;\;\beta_{4}t_{i}+\;\;\;\beta_{i}+e_{it} \end{array} \end{array}$$ (2)

where $\beta_{0}^{*}=$$\beta_{0}+4.61\beta_{1}$ and $\beta_{2}=-\beta_{1}$.

Model 1 and Model 2 are equivalent provided that $\beta_{0}^{*}=\;\alpha_{0}$, $\beta_{1}=\;\alpha_{1}$, $\beta_{2}=$$\alpha_{2}$, and $\beta_{3}=\;\alpha_{3}$. The set of cross-equation equalities of parameters, $\beta_{1}=\;\alpha_{1}$ and $\beta_{2}=$$\alpha_{2}$, and the restriction in equation (2) given by $\beta_{2}=-\beta_{1}$ reduces to a restriction on the parameters in equation (1) given by $\alpha_{2}=-\alpha_{1}$ or $\alpha_{1}+\alpha_{2}=0$.

Econometrically, Model 2 is thus a restricted form of Model 1 requiring price and income elasticity parameters to be equal. It means that if price and income increase by the same proportion, consumption will remain unchanged. Theoretically, it is equivalent to claiming that the indirect utility function is homogeneous with degree zero in prices and income—if prices and income are multiplied by a given constant, the same bundle of consumption maximizes utility. So, there is no money illusion and one would not expect any change in consumption. For price increases to induce a reduction in consumption, the price must increase by a larger proportion than income.

In the unrestricted Model (1), in contrast, consumption can reduce when $\left|\alpha_{1}\right|>\left|\alpha_{2}\right|$, even if price increases at the same rate as or at a lower rate than income provided that the difference in the absolute values of price elasticity and income elasticity is sufficiently large. Consumption can also reduce when $\left|\alpha_{1}\right|<\left|\alpha_{2}\right|$, provided that the price increase is sufficiently large to outweigh the effect of income growth. Thus, the net effect of price and income increases on consumption depends on the relative strength of the effects of price and income changes and is given by the sum $\Delta lnC_{it}=\;\alpha_{1}\Delta lnP_{it}+\alpha_{2}\Delta lnY_{it}$, where Δ stands for change in the corresponding variable.

The merit of using the restricted form is that by using a linear combination of log of price and log of income variables, $lnP_{it}-lnY_{it}$, Model 2 becomes a more parsimonious specification over Model 1. It requires only to test whether $\beta_{1}<0$ in Model 2 as opposed to jointly testing $\alpha_{1}<0$ and $\alpha_{2}>0$ along with $\alpha_{2}=-\alpha_{1}$ to ascertain the changes in consumption in response to both price and income changes.

Besides, the unrestricted Model 1 assumes homogeneous effects of price ($\alpha_{1})$ and income ($\alpha_{2})$ regardless of income levels or the stages of the tobacco epidemic, while there could be differential impacts. For example, $\alpha_{1i}$ and $\alpha_{2i}$ varying across *i* where *i* = income classification or the stages of the tobacco epidemic. Alternatively, Model 2 may have advantages when the linear combination $lnP_{it}-lnY_{it}$ sufficiently takes account of the systematic differences in both $\alpha_{1i}$ and $\alpha_{2i}$. This is particularly relevant when we pool different countries together that are less homogeneous, such as HICs and LMICs. Hence, we estimated each equation separately for HICs and LMICs.

The linear combination in Model 2, however, assigns equal strength to the effects of price and income changes, which may create model specification bias and is therefore subject to empirical validation.

Using global data on cigarette consumption, price, income, and relevant tobacco control measures corresponding to 169 countries for the period from 2007 to 2016, this study estimated the price elasticity (using Model 1) and affordability elasticity (using Model 2) of cigarette consumption by country income classification using country-specific fixed effects model for panel data in Stata 15. A complete list of countries included in the analysis is provided in Supplementary Appendix B. Robust standard errors of the estimated coefficients were obtained after adjustment for intracluster correlation of disturbances within countries. The assessment of model performance is based on the comparison of within *R*^2^ that indicates the power of each model in explaining within-country variation in cigarette consumption. We tested whether the abovementioned restrictions on the parameters of Model 1 and Model 2 are valid.

Finally, we explored the implication of using affordability elasticity and price elasticity (along with income elasticity) in evaluating and projecting the effectiveness of tobacco control policy through tax and price increases based on the following steps of a simulation exercise.

1. For a desired reduction in cigarette consumption by *C*_d_% when income growth is *Y*%, the required percentage increase in price would be given by *P*% = (*C*_d_% − *Y*% × *α*_2_)/*α*_1_ based on Model 1.
2. For the same reduction in cigarette consumption and income growth, the required percentage increase in RIP would be given by RIP% = (*C*_d_%/*β*_1_) and the required percentage increase in price would be given by *P*% = RIP% + *Y*% based on Model 2.
3. Given an initial tax rate of *t*% of price, the required percentage increase in tax per unit of cigarette consumption would be given by *T*% = *P*%/*t*% under the assumption that tax increase is fully passed on to price increase. The extent of the pass-through may, however, vary considerably across countries over time depending on the profit maximization strategy of tobacco companies largely driven by tobacco product market concentration and tobacco tax structure.^1^ When price increases at a higher rate than that warranted by a given tax increase due to overshifting by the industry, tobacco consumption decreases by a larger amount than it would be under a full pass-through scenario. In contrast, when price increases at a lower rate than that warranted by a given tax increase due to the absorption of a tax increase by the industry, tobacco consumption decreases by a smaller amount compared to the case of a full pass-through. Thus, overshifting can enhance the intended public health benefit of a tax increase, while undershifting can diminish it. The simulation exercise in this study sets these two scenarios aside to simplify the illustration of the application of affordability elasticity and price elasticity in tobacco tax policy evaluation without loss of generality.
4. The actual consumption effect of the tax increase informed by affordability elasticity would be given by *C*_a_% = *P*% (from step 2) × *α*_1_ + *Y*% × *α*_2_ when Model 1 is valid and *C*_a_% = RIP% (from step 2) × *β*_1_ when Model 2 is valid.
5. The revenue effect of the tax increase informed by affordability elasticity would be given by *R*% = *T*% (using affordability elasticity in step 3) + *C*_a_% (from step 4).

## Results

The results of the estimation of Model 1 (that estimates both price and income elasticity) and Model 2 (that estimates affordability elasticity) are presented in Table 1. The results can be summarized as follows:

1. Price elasticity estimates of per adult cigarette retail sale in Model 1 are negative and statistically significant for both HICs and LMICs.
2. The income elasticity estimate from Model 1 is positive and statistically significant for LMICs, while negative and statistically insignificant for HICs.
3. The affordability elasticity estimates from Model 2 are negative and statistically significant for both HICs and LMICs.
4. The restriction of the equality of the absolute values of the price and income elasticity estimates in Model 1 is rejected for HICs (*p* = .0493). The equivalence is not rejected for LMICs (*p* = .2198). It indicates that the restriction imposed on estimating affordability elasticity in Model 2 is not valid in the case of HICs. It is valid for LMICs only.
5. Models 1 and 2 perform equally well as reflected in the equal within *R*^2^ values for both HICs and LMICs.

**Table 1.** Fixed Effects Estimates of Per Adult Cigarette Retail Sale, 2007–2016

	HICs		LMICs	
	Model 1	Model 2	Model 1	Model 2
Log of cigarette price	−0.360^***^ (−3.65)		−0.212^**^ (−3.12)	
Log of per capita GDP	−0.157 (−0.84)		0.319^***^ (4.07)	
Log of relative income price		−0.171^**^ (−2.79)		−0.207^***^ (−4.93)
Population aged 15–64, % of total	0.040 (1.85)	0.0370 (1.61)	0.020 (1.49)	0.012 (0.89)
Population female, % of total	0.089^**^ (3.42)	0.096* (2.35)	−0.092 (−0.68)	−0.092 (−0.66)
Unemployment, % of labor force	−0.020^**^ (−2.90)	−0.014* (−2.43)	−0.003 (−0.70)	−0.002 (−0.52)
Lagged composite MPOWER score	−0.0004 (−0.00)	−0.0032 (−0.67)	−0.005* (−2.47)	−0.005* (−2.12)
Constant	2.471 (0.90)	0.262 (0.09)	7.528 (1.06)	10.79 (1.51)
Country-specific fixed effects	Yes	Yes	Yes	Yes
Country-specific trends	Yes	Yes	Yes	Yes
*F* test: $\alpha_{1}+\alpha_{2}=0$				
*p* value	.0493	—	.2198	—
Within *R*^2^	0.91	0.91	0.84	0.84
Number of countries	45	45	124	124
Observations	400	400	1103	1103

GDP = gross domestic product; HICs = high-income countries; LMICs = low- and middle-income countries.

The *t* statistics of the estimates are in parentheses. The regressions controlled for country-specific fixed effects and trends in per capita cigarette sales. The country-specific fixed effects and trend estimates were suppressed for the brevity of the presentation.

*p < .05, **p < .01, ***p < .001.

While the affordability elasticity estimates are comparable between HICs and LMICs, the price elasticity estimates are lower for LMICs than HICs and relatively low in comparison with available country-specific estimates.^2^ We are cognizant that we obtained an average of country-level estimates based on a within-country variation of the variables concerned and that global data tend to yield smaller estimates.^2,21^ We should, however, exercise caution in interpreting the average estimates for a group of countries which can deviate from the estimates obtained from country-level studies due to a mix of multiple factors. First, averages may be subject to the influence of outliers present in global data. Second, there may not be enough variation in prices in some countries to identify the effect of price changes on cigarette sales. For example, due to infrequent tax increases, prices remained invariant for the period under observation in China which is the top cigarette-consuming country in the world. It in turn led to increasing affordability and consumption of cigarettes in China.^22^ Third, countries subject to downward substitution to cheaper cigarettes in the presence of increasing prices may not see a decrease in sales or may even experience an increase in sales.^23^

Fourth, the low estimate of price elasticity for LMICs may partly be driven by the quality of data on cigarette sales and prices that are not routinely available for each year in all LMICs. It often requires modeling to fill in data gaps using interpolation or extrapolation from available datapoints that can smooth out time-series observations and cause loss of variability. In the present analysis, data were taken from the Euromonitor International database which provides actual data on cigarette sales and prices for 90 countries obtained from in-country sources. For the rest of the countries, that are predominantly LMICs, data are modeled. While the measurement error in cigarette sales (the dependent variable) in the Euromonitor International database does not bias the regression coefficients, the measurement error in the price variable (explanatory variable) is expected to make the absolute value of the price elasticity estimate biased downward. We ran a sensitivity analysis of the global price, income, and affordability elasticity estimates applying restriction on the inclusion of countries in the analysis based on the availability of actual data as opposed to modeled data on price and sales. The results are presented in Supplementary Appendix C, Table C1. The analysis suggests that the price elasticity estimates are insensitive to the exclusion of modeled data on cigarette sales and prices, while the income and affordability elasticity estimates may vary.

Finally, the country-level studies are not available for all countries included in this global study. The studies estimating price and income elasticity of cigarette demand in LMICs began to be published on a global scale in the 2000s onward and are not exhaustive yet in covering all LMICs. A global average price elasticity as low as −0.212 for LMICs in contrast to −0.360 for HICs may very well indicate that tax and price measure has not worked as effectively in all LMICs as it had in most HICs in the recent decade. As a matter of fact, taxation has been the least implemented measure of MPOWER policies in the member states in terms of population coverage.^19^

The difference in the test results between LMICs and HICs on the restriction of the equality of the absolute values of the price and income elasticity in Model 1 is likely related to the trajectory of economic growth in these two sets of countries. Prior to the economic downturn and recession setting in 2008–2009, rapid economic growth was experienced worldwide. Please see Table 2 for trend growth rates in per capita GDP by country income groups and pre- and post-recession era in 2002–2006 and 2007–2016, respectively. In both periods, HICs experienced sluggish or no economic growth with no discernible effect on cigarette consumption. It resulted in statistically insignificant estimates of the income elasticity of cigarette consumption for HICs while the effectiveness of price increase in reducing cigarette consumption was still at work. The rejection of the equivalence of price and income elasticity in HICs is partly attributable to an almost complete absence of economic growth in HICs. Besides, the negative sign on the income elasticity estimate for HICs may suggest that cigarettes are likely turning into inferior products so that per capita cigarette sale is going down with a higher income level in HICs. It is likely that higher income is linked to higher education and better awareness about the harms of smoking that induces people to abstain from smoking.

**Table 2.** Trend Growth Rates (%) of Per Capita GDP, 2002–2016

Period	Global	HICs	LMICs
2002–2016	2.31 (.000)	1.13 (.000)	2.75 (.000)
2002–2006	3.69 (.000)	3.17 (.000)	3.87 (.000)
2007–2016	1.68 (.000)	0.59 (.000)	2.07 (.000)
Number of countries	169	45	124

GDP = gross domestic product; HICs = high-income countries; LMICs = low- and middle-income countries.

Authors’ estimation using fixed effects regression ln*Y*_*it*_ = *a*_0_ + *a*_1_*t*_*i*_ + *α*_*i*_ + *u*_*it*_ (where *Y* = income and *t* = year) based on time-series cross-section data compiled for 169 countries. The estimates of the coefficient of the time variable are presented in this table and represent the annual trend growth rate of income. The *p* values of the estimates are in parentheses.

Economic growth was more pronounced in LMICs than HICs even in the period after the global recession. Rapid income growth makes the effect of income on consumption more discernible which seems to be driving the positive and statistically significant income elasticity estimates. Comparing the absolute values of price and income elasticity estimates for LMICs in Table 1, it appears that the individual effects of income and price changes on cigarette consumption were equally strong resulting in the “non-rejection” of the equality of price and income elasticity estimates in absolute terms.

The coefficient of the MPOWER score is not statistically significant for HICs for at least two major reasons. First, we controlled for country-specific year trends along with country-specific fixed effects. Unlike prices and thus RIPs, there may be limited within-country over time variations in HICs in the recent past to allow for the identification of the effect of MPOWER scores for these countries. In the absence of significant within-country variation, MPOWER scores control for the between-country variations in the tobacco control policy environment only. Second, the MPOWER score does not measure the full scale of the WHO FCTC implementation. It is only the strength of implementation of tobacco tax and price measure (*R*) that is reflected in the changes in prices and RIP. The statistically significant price and affordability elasticity estimates attest to the fact that the implementation of *R* measure has been effective in reducing cigarette consumption globally. We do not claim in this article that we have estimated the effect size of WHO FCTC implementation.

## Simulation

Using the price, income, and affordability estimates presented in Table 1, we ran a policy simulation to show the required tax and price increases to achieve the desired reduction in cigarette consumption by 10% for a representative HIC and an LMIC. The income growth rate was assigned 0.59% for the HIC and 2.1% for the LMIC, based on the corresponding trend estimates in Table 2 for the period of 2007–2016. The initial tax rate was assumed 50% of the current retail price.

As given in Table 3, the required price increase calculated using affordability elasticity is much larger for the HICs and similar for the LMICs than those indicated by the price and income elasticities—compare 59.1% versus 27.8% for HICs and 50.4% versus 50.3% for LMIC. The required tax increases show the same pattern.

**Table 3.** Results of Simulation of Tax and Price Increase Required to Attain the Desired Reduction in Tobacco Consumption

	HICs	LMICs
Price elasticity	−0.360	−0.212
Income elasticity	−0.157	0.319
Affordability elasticity	−0.171	−0.207
Desired change in cigarette consumption	−10.0%	−10.0%
Change in income	0.59%	2.1%
Required change in price using price and income elasticities^i^	27.8%	50.3%
Required change in RIP using affordability elasticity^ii^	58.5%	48.3%
Required change in price using affordability elasticity^iii^	59.1%	50.4%
Initial tax rate (% of price)	50%	50%
Required tax increase (per unit of cigarette consumption)		
Using both price and income elasticities^iv^	56%	101%
Using affordability elasticity^v^	118%	101%
Consumption effect of a tax change informed by affordability elasticity		
Using price and income elasticities^vi^	−21%	−10%
Using affordability elasticity^vii^	−10%	−10%
Revenue effect of a tax change informed by affordability elasticity		
Using price and income elasticities^viii^	34%	91%
Using affordability elasticity^ix^	108%	91%

HICs = high-income countries; LMICs = low- and middle-income countries.

i. Required change in price based on price and income elasticities = (Desired change in consumption – Change in income × Income elasticity)/Price elasticity.

ii. Required change in Relative Income Price (RIP) based on affordability elasticity = Desired change in consumption/Affordability elasticity.

iii. Required change in price based on affordability elasticity = Desired change in consumption/Affordability elasticity + Change in income.

iv. Required change in tax based on price and income elasticities = Required change in price based on price and income elasticities/Initial tax rate, assuming full pass-through of tax increase.

v. Required change in tax based on affordability elasticity = Required change in price based on affordability elasticity/Initial tax rate, assuming full pass-through of tax increase.

vi. Consumption effect of a tax change (informed by affordability elasticity) based on price and income elasticities = Required change in price based on affordability elasticity × Price elasticity + Change in income × Income elasticity.

vii. Consumption effect of a tax change (informed by affordability elasticity) based on affordability elasticity = Required change in RIP based on affordability elasticity × Affordability elasticity.

viii. Revenue effect of a tax change (informed by affordability elasticity) based on price and income elasticities = Required change in tax based on affordability elasticity + Consumption effect of a tax change (informed by affordability elasticity) based on price and income elasticities.

ix. Revenue effect of a tax change (informed by affordability elasticity) based on affordability elasticity = Required change in tax based on affordability elasticity + Consumption effect of a tax change (informed by affordability elasticity) based on affordability elasticity.

Given that the hypothesis of the equality of the absolute values of price and income elasticity parameters was rejected for HICs while not rejected for LMICs (Table 1), it can be argued that the application of the affordability elasticity parameter to HICs may overstate the required tax increase. The question is what difference this overstatement would make to the policy makers. The simulated effects on consumption and revenue of the tax increase informed by affordability elasticity, given in Table 3, show that the decrease in consumption would be much larger and the increase in revenue would be much smaller than intended by the affordability elasticity parameter in case of HICs, while the predictions remain similar in case of LMICs. This is because while the responsiveness of consumption to tax and price increases predicted from affordability elasticity offers a close approximation to the reality in LMICs, it can misrepresent the reality in HICs where cigarette consumption is not sensitive to income growth as reflected in the statistically insignificant income elasticity estimate for HICs (Table 1).

## Discussion

Based on global panel data on cigarette sales, the results of this article conform to the existing evidence that the price elasticity of cigarette consumption is negative and statistically significant, which implies that tobacco tax increases that induce tobacco price increases reduce tobacco consumption. The results also confirm that in measuring the effectiveness of tobacco tax and price increases in reducing tobacco consumption, it is important to consider the effect of income growth that can offset (partly or fully) the effect of tax and price increases. In other words, in the presence of rapid income growth, the tax and price increases required to effectively reduce tobacco consumption at the population level would be larger than the increases required under conditions of sluggish or no economic growth.

The major contribution of this article lies in indicating that in the countries experiencing fast income growth of current and potential consumers of tobacco products, affordability elasticity can provide a reasonable benchmark for tax and price increase necessary to effectively reduce affordability and consumption of tobacco. This is more likely the case in LMICs. In contrast, the effect of income changes may not be discernible in a high-income setting with a plateaued income trajectory. In these circumstances, separate price and income elasticity estimates may prove more useful to make an accurate prediction of the effect of tax and price increases on tobacco consumption and revenue. To our knowledge, this is the first attempt to obtain global estimates of price and income, and affordability elasticity and to test the validity of using one versus the other.

For researchers estimating and applying elasticities of tobacco demand in the tobacco control policy framework in a country, an important recommendation follows from this study that they estimate both price elasticity (in conjunction with income elasticity) and affordability elasticity using two separate models from available data. The next step is to test the equality of the absolute values of the price and income elasticity estimates. If the equality is not rejected, they can apply affordability elasticity to offer a lower bound to the required tax and price increase for the desired reduction in consumption. If equality is rejected, affordability elasticity can misinform the policy making process by overstating the required tax and price increase. In that case, price and income elasticity estimates would be more reliable parameters for guiding policy decisions.

Affordability in general is a very useful concept in tobacco control, separate from affordability elasticity. Even if the use of affordability elasticity is found to be far from ideal in specific circumstances, tobacco control advocates can effectively use the term “affordability” conceptually when talking to policy makers. In fact, it is important to explain the combined effects of simultaneous changes in price and income to policy makers and the relevance of adjusting tax and price levels in tandem with income growth and inflation. Price as a concept resonates well and hence it seems to be the right starting point with most individuals, such as policy makers. Nearly everyone can relate to it. But then, we need to add the additional concepts of income change and affordability to add critical nuance. Most importantly, it reinforces why we need a built-in policy mechanism that can facilitate regular price changes; otherwise, policy makers have a challenging time understanding why they must keep raising prices, setting aside the regular adjustment for inflation. Until and unless the process of regular adjustment of tax for inflation and income growth is integrated into the tobacco tax policy mechanism universally, tobacco control advocates will have to fight an incessant battle against the tobacco epidemic on this front.

The study is not free from its limitations which can be addressed in future research. First, the study stayed away from linking cigarette price increases to tax increases at the country level which can be mediated by industry responses to tax increases to a great extent and hence fell short of making a direct evaluation of the effectiveness of tax increases in reducing cigarette demand at the global level. While industry responses to tax policy changes can be effectively captured in country-level analysis, a global analysis would be far more complicated because country-specific experiences may not necessarily be generalizable and comparable measures are hardly available. The second limitation is the lack of adequate data for LMICs. We have worked with modeled data for many LMICs. With a more complete dataset for LMICs, the results might look somewhat different from what we obtained in the study. However, the primary purpose of estimating price, income, and affordability elasticity in this article was to demonstrate the key relationships in the determination of cigarette demand and we used a global dataset for illustrating these relationships. The quality or availability of data and its implications for the unbiased and precise estimates of elasticities were not the focus of this exercise.

## Conclusions

A model estimating affordability elasticity is a restricted version of a model that estimates price and income elasticities. Nonetheless, affordability elasticity may prove to be a useful parameter to explain and predict the sensitivity of tobacco users to tax and price policy changes under conditions of robust economic growth, which are more likely to be observed in countries with initial low- or middle-income setting. It can provide a reasonable benchmark for tobacco tax and price increase necessary to effectively reduce affordability and consumption of tobacco, which can form a basis for building systematic tax and price increases into the tobacco tax policy mechanism.

## Supplementary Material

A Contributorship Form detailing each author’s specific involvement with this content, as well as any supplementary data, is available online at https://academic.oup.com/ntr

ntaa134_suppl_Supplementary_File

ntaa134_suppl_Supplementary_Taxonomy_Form
