# Supplementary material for: Price, Income, and Affordability as the Determinants of Tobacco Consumption: A Practitioner’s Guide to Tobacco Taxation
Source: Nicotine Tob Res. 2020 Jul 22;23(1):40–7. doi: 10.1093/ntr/ntaa134 (PMC7789936; doi:10.1093/ntr/ntaa134)
Supplement: ntaa134_suppl_Supplementary_File [file ntaa134_suppl_supplementary_file.docx]

## Appendix A: Data and Measures

### **Cigarette consumption variable**

The primary outcome variable is annual cigarette consumption per adult (in number of cigarette sticks) calculated by dividing total retail volume of cigarettes (available from the Euromonitor International Database) by the size of the adult population ages 15 and above (available from the US Census Bureau International Database) in each country and year from 2007 through 2016[8,9]. The price, income and affordability elasticity estimates obtained in this paper based on per adult cigarette consumption refer to the elasticity of total adult cigarette consumption accounting for both smoking prevalence and smoking intensity among adults in the following way:

Total annual cigarette consumption among adults = Number of adult smokers X Number of cigarettes smoked per adult smoker annually (adult smoking intensity)

where, Number of adult smokers = Adult smoking prevalence X Total adult population

Thus, Per adult cigarette consumption = Total annual cigarette consumption among adults/Total adult population = Adult smoking prevalence X Adult smoking Intensity

Using natural log on both sides of the equation,

ln(Per adult cigarette consumption) = ln(Adult smoking prevalence) + ln(Adult smoking Intensity)

Taking partial derivative with respect to P,

d ln(Per adult cigarette consumption)/dP = d ln(Adult smoking prevalence)/dP + d ln(Adult smoking Intensity)/dP

or, Total elasticity = Elasticity of smoking prevalence + Elasticity of smoking intensity

We preferred to use per adult cigarette consumption as an outcome measure because the purpose of tobacco tax and price increases is to reduce both smoking prevalence and smoking intensity. This is a standard practice in the measurement of price and income sensitivity of tobacco demand. Using consumption per adult smoker would result in an estimate of only the elasticity of smoking intensity, which would be a biased estimate of total elasticity.

However, we recognize that the initial smoking prevalence reflects the stage of smoking epidemic and the omission of country level stage of smoking epidemic may bias the estimate of price elasticity of smoking prevalence. Because, the countries in the earlier stages of the epidemic are expected to experience increase in smoking prevalence, while the countries in the final stage of the epidemic are expected to see declining smoking prevalence. As a result, omission of initial smoking prevalence may result in a downward bias of the elasticity estimates in the countries in the final stage and upward bias in the countries in the earlier stages of the smoking epidemic[8].

In the country-level fixed effects panel data regression model used for analysis in this paper, controlling for initial smoking prevalence or the stage of the smoking epidemic would be redundant as it is merged in the country-level fixed effects. Besides, any trend in smoking prevalence due to factors other than those controlled for in the model would be captured in the country-specific trend variable. Hence, we can claim that the disparities in smoking prevalence across countries has not caused any bias in the price, income and affordability elasticity estimates.

### **The price variable**

The average price per pack of 20 cigarettes was calculated by dividing the total retail value by the annual retail volume, both reported in the Euromonitor International Ltd Database. The nominal price variable for each country over time was adjusted for inflation and converted into 2016 constant prices (real prices) using the consumer price indices (CPIs) for respective countries. For cross-country comparability of prices, the real prices were converted into international dollars using 2016 purchasing power parity (PPP) conversion factors for respective countries. The CPI and the PPP conversion factors were obtained from the World Economic Outlook (WEO) Database of the International Monetary Fund[10].

## **The income variable**

We used per capita gross domestic product (PGDP) as the income variable adjusted for inflation and purchasing power parity (PPP) using annual GDP deflators and 2016 PPP conversion factors for respective countries and expressed in 2016 constant prices like the price variable. The PGDP data in current local currency units were drawn from the World Bank World Development Indicators (WDI) Database[11].

### **Affordability measure**

Following Blecher and Van Walbeek, the affordability of cigarettes was measured as the percentage of income required to purchase 100 packs of 20 cigarettes[4]. The price and income variables as described above were used to construct the RIP variable.

### **Other control variables**

We controlled for aggregated country-level demographic and macroeconomic characteristics in the analysis. These variables include the percentage of working age persons ages 15-64 (available from the US Census Bureau International Database), the percentage of females in the total population and percentage of the unemployed in the total labor force (available from the WDI Database[9,11].

### **Composite MPOWER scores**

In addition, we used the composite MPOWER scores to control for the tobacco control policy environment reflected in the level of implementation of the WHO FCTC. The WHO FCTC is the first international public health treaty under the auspices of the World Health Organization, which was adopted in 2003 and came into force in 2005 to protect nations from the devastating health, economic, social and environmental consequences of tobacco use and exposure to tobacco smoke[12,13]. In 2008, WHO introduced the package of six best-practice and cost-effective demand-reduction tobacco control policy measures contained in the WHO FCTC—monitor tobacco use (M), protect people from tobacco smoke (P); offer help to quit tobacco use (O); warn about the dangers of tobacco (W) with two sub-components, health warnings (W1) and mass media (W2); enforce bans on tobacco advertising, promotion and sponsorship (E); and raise taxes on tobacco products (R)—collectively known as MPOWER[14]. The country-specific MPOWER scores were systematically measured and reported in the bi-annual WHO Report on the Global Tobacco Epidemic since 2007[14-19]. The individual policy score ranges from 1 to 4 for M, and from 1 to 5 for P, O, W1, W2, E, and R. Score 1 indicates missing data, while scores 2 and above represent the strength of implementation. The higher the score, the stronger the level of implementation.

For present analysis, score 1 was set to missing value and scores 2, 3, 4 and 5 were recoded as scores 1, 2, 3 and 4 respectively maintaining the order of the strength of implementation. The composite MPOWER score was obtained using the sum of the individual scores of the seven indicators M, P, O, W1, W2, E, and R. Since the MPOWER scores were not reported in the interim years (2009, 2011, 2013, 2015) between two consecutive reports, we imputed the scores from the previous years to those missing years. Following He and colleagues, it was assumed that the level of implementation did not change significantly in the year immediately following the reporting year. Since it usually takes time to discern the policy impact on the outcome of interest, we used lagged values of the MPOWER score.

**Country income classification**

Countries were categorized into low-and-middle-income countries (LMICs) and high-income countries (HICs) based on World Bank economic classification in 2016 available in the WDI database[11].

The summary statistics of the outcome measure and control variables used in the regression analysis are provided in Table A1 below by country income status and year of observation. The mean values are reported for 2008 through 2016 for all variables except the composite MPOWER score which is used with one-year lag. Thus, we lose observations on all variables except MPOWER measure in the estimation sample for 2007.

| **Table A1: Sample means of outcome measure and control variables by country income status, 2008-2016.** | | | | | | | | | | | |
| --- | --- | --- | --- | --- | --- | --- | --- | --- | --- | --- | --- |
| **HICs (N = 45)** | 2008 | 2009 | | 2010 | | 2011 | 2012 | 2013 | 2014 | 2015 | 2016 |
| Per adult annual cigarette sale (sticks) | 1,469 | 1,394 | | 1,314 | | 1,257 | 1,220 | 1,152 | 1,101 | 1,076 | 1,060 |
| Cigarette price per pack of 20 (2016 $PPP) | 5.42 | 5.61 | | 5.85 | | 6.06 | 6.27 | 6.54 | 6.77 | 7.01 | 7.23 |
| Per capita GDP (2016 $PPP) | 37149 | 35,287 | | 35,859 | | 36,066 | 36,547 | 36,734 | 36,871 | 37,623 | 38,099 |
| Relative Income Price (%) | 1.3 | 1.5 | | 1.6 | | 1.6 | 1.7 | 1.7 | 1.8 | 1.8 | 1.9 |
| Population aged 15-64, % of total | 68.9 | 68.9 | | 68.9 | | 68.8 | 68.6 | 68.4 | 68.2 | 68.0 | 67.8 |
| Population female, % of total | 49.1 | 49.0 | | 49.0 | | 48.9 | 48.9 | 48.9 | 48.9 | 48.9 | 48.9 |
| Unemployment, % of labor force | 5.6 | 7.7 | | 8.2 | | 8.0 | 8.3 | 8.3 | 7.7 | 7.1 | 6.7 |
| Lagged composite MPOWER score | 16.7 | 16.7 | | 17.9 | | 17.9 | 18.2 | 18.2 | 18.4 | 18.4 | 19.7 |
| **LMICs (N = 124)** | 2008 | | 2009 | | 2010 | 2011 | 2012 | 2013 | 2014 | 2015 | 2016 |
| Per adult annual cigarette sale (sticks) | 890 | | 870 | | 857 | 833 | 817 | 787 | 750 | 724 | 705 |
| Cigarette price per pack of 20 (2016 $PPP) | 3.83 | | 3.99 | | 3.77 | 4.10 | 4.15 | 4.21 | 3.65 | 3.72 | 3.85 |
| Per capita GDP (2016 $PPP) | 7,544 | | 7,423 | | 7,599 | 7,779 | 8,038 | 8,249 | 8,375 | 8,374 | 8,453 |
| Relative Income Price (%) | 7.9 | | 7.7 | | 7.4 | 7.1 | 6.7 | 6.5 | 6.4 | 6.5 | 6.6 |
| Population aged 15-64, % of total | 60.5 | | 60.7 | | 61.0 | 61.3 | 61.5 | 61.8 | 61.9 | 62.1 | 62.3 |
| Population female, % of total | 50.3 | | 50.3 | | 50.3 | 50.3 | 50.3 | 50.2 | 50.2 | 50.2 | 50.2 |
| Unemployment, % of labor force | 8.2 | | 8.5 | | 8.6 | 8.3 | 8.2 | 8.2 | 8.2 | 8.2 | 8.0 |
| Composite MPOWER score | 12.2 | | 12.2 | | 13.4 | 13.4 | 14.3 | 14.2 | 15.5 | 15.5 | 15.7 |

Note: The changes in the mean of country-specific per capita GDP in 2016 PPP$ reported in this table for HICs and LMICs are not be interpreted as the average trend in per capita GDP for each group of countries. The average trend in per capita GDP by country income group were estimated by fitting trend regression to country-level data and the results were presented in Table 2 for two time intervals of 2002-2006 and 2007-2016.

## Appendix B: List of countries included in the analysis

|  | **Full Sample** | **Restricted Sample** |
| --- | --- | --- |
| **High-Income Countries** | | |
| 1. | Australia | Australia |
| 2. | Austria | Austria |
| 3. | Bahamas | Bahamas |
| 4. | Bahrain | Bahrain |
| 5. | Barbados | Barbados |
| 6. | Belgium | Belgium |
| 7. | Canada | Canada |
| 8. | Chile | Chile |
| 9. | Czech Republic | Czech Republic |
| 10. | Denmark | Denmark |
| 11. | Estonia | Estonia |
| 12. | Finland | Finland |
| 13. | France | France |
| 14. | Germany | Germany |
| 15. | Greece | Greece |
| 16. | Hungary | Hungary |
| 17. | Iceland | Iceland |
| 18. | Ireland | Ireland |
| 19. | Israel | Israel |
| 20. | Italy | Italy |
| 21. | Japan | Japan |
| 22. | Kuwait |  |
| 23. | Latvia | Latvia |
| 24. | Lithuania | Lithuania |
| 25. | Netherlands | Netherlands |
| 26. | New Zealand | New Zealand |
| 27. | Norway | Norway |
| 28. | Oman |  |
| 29. | Poland | Poland |
| 30. | Portugal | Portugal |
| 31. | Qatar |  |
| 32. | Saudi Arabia |  |
| 33. | Seychelles | Seychelles |
| 34. | Singapore | Singapore |
| 35. | Slovakia | Slovakia |
| 36. | Slovenia | Slovenia |
| 37. | South Korea | South Korea |
| 38. | Spain | Spain |
| 39. | Sweden | Sweden |
| 40. | Switzerland | Switzerland |
| 41. | Trinidad and Tobago | Trinidad and Tobago |
|  | **Full Sample** | **Restricted Sample** |
| 42. | United Arab Emirates | United Arab Emirates |
| 43. | United Kingdom | United Kingdom |
| 44. | Uruguay | Uruguay |
| 45. | USA | USA |
| **Low and Middle-Income Countries** | | |
| 1. | Afghanistan | Afghanistan |
| 2. | Albania | Albania |
| 3. | Algeria |  |
| 4. | Angola | Angola |
| 5. | Argentina | Argentina |
| 6. | Armenia |  |
| 7. | Azerbaijan | Azerbaijan |
| 8. | Bangladesh | Bangladesh |
| 9. | Belarus |  |
| 10. | Belize | Belize |
| 11. | Benin |  |
| 12. | Bolivia |  |
| 13. | Bosnia-Herzegovina | Bosnia-Herzegovina |
| 14. | Botswana |  |
| 15. | Brazil | Brazil |
| 16. | Bulgaria | Bulgaria |
| 17. | Burkina Faso | Burkina Faso |
| 18. | Burundi | Burundi |
| 19. | Cabo Verde | Cabo Verde |
| 20. | Cambodia | Cambodia |
| 21. | Cameroon | Cameroon |
| 22. | Central African Republic | Central African Republic |
| 23. | Chad | Chad |
| 24. | China |  |
| 25. | Colombia | Colombia |
| 26. | Comoros | Comoros |
| 27. | Congo, Democratic Republic |  |
| 28. | Congo, Republic | Congo, Republic |
| 29. | Costa Rica | Costa Rica |
| 30. | Côte d'Ivoire |  |
| 31. | Croatia | Croatia |
| 32. | Djibouti | Djibouti |
| 33. | Dominican Republic | Dominican Republic |
| 34. | Ecuador | Ecuador |
| 35. | Egypt | Egypt |
| 36. | El Salvador | El Salvador |
| 37. | Equatorial Guinea | Equatorial Guinea |
| 38. | Eritrea | Eritrea |
|  | **Full Sample** | **Restricted Sample** |
| 39. | Ethiopia |  |
| 40. | Fiji | Fiji |
| 41. | Gabon |  |
| 42. | Gambia | Gambia |
| 43. | Georgia | Georgia |
| 44. | Ghana |  |
| 45. | Grenada | Grenada |
| 46. | Guatemala | Guatemala |
| 47. | Guinea |  |
| 48. | Guinea-Bissau |  |
| 49. | Guyana | Guyana |
| 50. | Haiti | Haiti |
| 51. | Honduras |  |
| 52. | India | India |
| 53. | Indonesia |  |
| 54. | Iran, Islamic Republic of |  |
| 55. | Iraq |  |
| 56. | Jamaica | Jamaica |
| 57. | Jordan | Jordan |
| 58. | Kazakhstan | Kazakhstan |
| 59. | Kenya |  |
| 60. | Kyrgyzstan | Kyrgyzstan |
| 61. | Laos |  |
| 62. | Lebanon | Lebanon |
| 63. | Lesotho | Lesotho |
| 64. | Liberia | Liberia |
| 65. | Libya |  |
| 66. | Macedonia |  |
| 67. | Madagascar | Madagascar |
| 68. | Malawi | Malawi |
| 69. | Malaysia | Malaysia |
| 70. | Maldives | Maldives |
| 71. | Mali | Mali |
| 72. | Mauritania | Mauritania |
| 73. | Mauritius | Mauritius |
| 74. | Mexico | Mexico |
| 75. | Moldova | Moldova |
| 76. | Mongolia | Mongolia |
| 77. | Montenegro | Montenegro |
| 78. | Morocco |  |
| 79. | Mozambique | Mozambique |
| 80. | Myanmar | Myanmar |
| 81. | Namibia |  |
|  | **Full Sample** | **Restricted Sample** |
| 82. | Nepal | Nepal |
| 83. | Nicaragua | Nicaragua |
| 84. | Niger | Niger |
| 85. | Nigeria |  |
| 86. | Pakistan | Pakistan |
| 87. | Panama |  |
| 88. | Papua New Guinea | Papua New Guinea |
| 89. | Paraguay |  |
| 90. | Peru | Peru |
| 91. | Philippines | Philippines |
| 92. | Romania | Romania |
| 93. | Russian Federation | Russian Federation |
| 94. | Rwanda | Rwanda |
| 95. | Samoa | Samoa |
| 96. | Sao Tomé e Príncipe |  |
| 97. | Senegal | Senegal |
| 98. | Serbia | Serbia |
| 99. | Sierra Leone | Sierra Leone |
| 100. | Solomon Islands | Solomon Islands |
| 101. | South Africa | South Africa |
| 102. | South Sudan |  |
| 103. | Sri Lanka |  |
| 104. | St Lucia | St Lucia |
| 105. | St Vincent and the Grenadines | St Vincent and the Grenadines |
| 106. | Sudan | Sudan |
| 107. | Suriname | Suriname |
| 108. | Tajikistan | Tajikistan |
| 109. | Tanzania | Tanzania |
| 110. | Thailand |  |
| 111. | Togo |  |
| 112. | Tonga | Tonga |
| 113. | Tunisia | Tunisia |
| 114. | Turkey | Turkey |
| 115. | Turkmenistan | Turkmenistan |
| 116. | Uganda | Uganda |
| 117. | Ukraine | Ukraine |
| 118. | Uzbekistan | Uzbekistan |
| 119. | Vanuatu | Vanuatu |
| 120. | Venezuela |  |
| 121. | Vietnam |  |
| 122. | Yemen | Yemen |
| 123. | Zambia |  |
| 124. | Zimbabwe | Zimbabwe |

Note: The countries were classified into high-income and low-and-middle-income status based on the World Bank Economic Classification as of 2016 (the final year of observation in data). The Full Sample was defined based on the availability of non-missing or modelled data on all the variables included in the regression analysis. The Restricted Sample excludes the countries with modelled data obtained from Euromonitor International.

## Appendix C: Sensitivity Analysis

We estimated Models 1 and 2 after exclusion of modeled data on cigarette sales and prices. The imputation of missing data is done at the country level by Euromonitor International. They do not provide country-specific details of the modelling procedure they use in-house for the imputation with the database that we can access through subscription. In the absence of adequate information on the background modelling procedure of imputation, we ran a sensitivity analysis of the price elasticity estimate by excluding the countries with modeled data in Restricted Sample 2 that also drops the countries showing positive association between price and cigarette sales as in Restricted Sample 1.

As shown in Table C1, the price elasticity estimates were found very close in the Full Sample and the Restricted Sample for both HICs and LMICs. The income elasticity estimate continued to be statistically insignificant in the Restricted Sample as in the Full Sample for HICs, while it increased in the Restricted Sample compared to the Full Sample for LMICs. The affordability elasticity estimate increased somewhat in the Restricted Sample compared to the Full Sample for both HICs and LMICs.

**Table C1: Fixed effects estimates of per adult cigarette retail sale, 2007-2016.**

|  | HICs | | | | LMICs | | | |
| --- | --- | --- | --- | --- | --- | --- | --- | --- |
|  | Full Sample | | Restricted Sample | | Full Sample | | Restricted Sample | |
|  | Model 1 | Model 2 | Model 1 | Model 2 | Model 1 | Model 2 | Model 1 | Model 2 |
| Log of cigarette price | -0.360^***^ |  | -0.351^***^ |  | -0.212^**^ |  | -0.234^*^ |  |
|  | (-3.65) |  | (-3.61) |  | (-3.12) |  | (-2.64) |  |
| Log of per capita GDP | -0.157 |  | -0.048 |  | 0.319^***^ |  | 0.466^*^ |  |
|  | (-0.84) |  | (-0.20) |  | (4.07) |  | (3.40) |  |
| Log of Relative Income Price |  | -0.171^**^ |  | -0.251^***^ |  | -0.207^***^ |  | -0.234^**^ |
|  |  | (-2.79) |  | (-4.28) |  | (-4.93) |  | (-3.06) |
| Population aged 15-64, % of total | 0.040 | 0.0370 | 0.042 | 0.037 | 0.020 | 0.012 | 0.034 | 0.031 |
|  | (1.85) | (1.61) | (1.90) | (1.55) | (1.49) | (0.89) | (0.04) | (1.61) |
| Population female, % of total | 0.089^**^ | 0.096^*^ | 0.060 | 0.047 | -0.092 | -0.092 | -0.180 | -0.146 |
|  | (3.42) | (2.35) | (1.26) | (1.04) | (-0.68) | (-0.66) | (-0.45) | (-0.35) |
| Unemployment, % of labor force | -0.020^**^ | -0.014^*^ | -0.021^**^ | -0.014^*^ | -0.003 | -0.002 | -0.005 | -0.006 |
|  | (-2.90) | (-2.43) | (-3.06) | (-2.50) | (-0.70) | (-0.52) | (-0.58) | (-0.55) |
| Lagged Composite MPOWER Score | -0.0004 | -0.0032 | -0.001 | -0.003 | -0.005^*^ | -0.005^*^ | -0.011^**^ | -0.010^*^ |
|  | (-0.09) | (-0.67) | (-0.11) | (-0.61) | (-2.47) | (-2.12) | (-0.003) | (-2.66) |
| Constant | 2.471 | 0.262 | 2.645 | 2.722 | 7.528 | 10.79 | 10.072 | 12.709 |
|  | (0.90) | (0.09) | (0.82) | (0.90) | (1.06) | (1.51) | (0.49) | (0.60) |
| Country-specific fixed effects | Yes | Yes | Yes | Yes | Yes | Yes | Yes | Yes |
|  |  |  |  |  |  |  |  |  |
| Country-specific trends | Yes | Yes | Yes | Yes | Yes | Yes | Yes | Yes |
|  |  |  |  |  |  |  |  |  |
| Within R^2^ | 0.91 | 0.91 | 0.91 | 0.90 | 0.84 | 0.84 | 0.84 | 0.84 |
| Number of countries | 45 | 45 | 38 | 38 | 124 | 124 | 58 | 58 |
| Number of observations | 400 | 400 | 342 | 342 | 1103 | 1103 | 522 | 522 |

Notes:

1. The *t* statistics of the estimates are in parentheses.

2. ^*^ *p* < 0.05, ^**^ *p* < 0.01, ^***^ *p* < 0.001.

3. The regressions controlled for country-specific fixed effects and trends in per capita cigarette sales. The country-specific fixed effects and trend estimates were suppressed for the brevity of presentation.

4. Full Sample refers to all countries with non-missing or modelled data on all variables required for regression analysis.

5. The Restricted Sample excludes the countries with modelled data obtained from Euromonitor International.
